# Development and Validation of a Model Including Distinct Vascular Patterns to Estimate Survival in Hepatocellular Carcinoma

**DOI:** 10.1001/jamanetworkopen.2021.25055

**Published:** 2021-09-13

**Authors:** Wen-Ping Lin, Kai-Li Xing, Jian-Chang Fu, Yi-Hong Ling, Shao-Hua Li, Wu-Shen Yu, Yong-Fa Zhang, Chong Zhong, Jia-Hong Wang, Zhi-Yuan Chen, Liang-He Lu, Wei Wei, Rong-Ping Guo

**Affiliations:** 1 Department of Hepatobiliary Oncology, Sun Yat-Sen University Cancer Center, Guangzhou, China; 2 State Key Laboratory of Oncology in South China, Collaborative Innovation Center for Cancer Medicine, Guangzhou, China; 3 Department of Pancreatobiliary Surgery, Sun Yat-Sen University Cancer Center, Guangzhou, China; 4 Department of Pathology, Sun Yat-Sen University Cancer Center, Guangzhou, China; 5 Department of General Surgery, Dongguan People’s Hospital, Southern Medical University, Dongguan City, China; 6 Department of Hepatic Surgery, Fudan University, Shanghai Cancer Center, Shanghai, China; 7 Department of Oncology, Shanghai Medical College, Fudan University, Shanghai, China; 8 Department of Hepatobiliary Surgery, The First Affiliated Hospital of Guangzhou University of Chinese Medicine, Guangzhou, China; 9 Department of Abdominal Surgery, Affiliated Cancer Hospital and Institute of Guangzhou Medical University, Guangzhou, China; 10 Department of Gastroenterology, Hunan Provincial People’s Hospital, The First Affiliated Hospital of Human Normal University, Changsha, China

## Abstract

**Importance:**

Because of tumor heterogeneity, traditional clinical variables remain insufficient to predict recurrence, which impairs long-term survival among patients undergoing radical hepatectomy for hepatocellular carcinoma (HCC). Vessels encapsulating tumor clusters (VETC) constitute a novel vascular pattern distinct from microvascular invasion (MVI), representing biological aggressiveness of HCC.

**Objective:**

To establish a model to estimate individualized recurrence-free survival (RFS) in HCC by integrating VETC and MVI.

**Design, Setting, and Participants:**

This prognostic study included 498 patients undergoing radical hepatectomy for HCC from 5 academic centers in China from January 1, 2013, to December 31, 2016, and consisted of 3 cohorts: training (243 [48.8%]), internal validation (122 [24.5%]), and external validation (133 [26.7%]). Follow-up was completed on March 30, 2020, and the data were analyzed from December 1 to 31, 2020.

**Exposures:**

VETC, MVI, tumor number, and maximum tumor size.

**Main Outcomes and Measures:**

The primary end point was RFS. The risk score for relative recurrence and nomogram for absolute RFS probability were derived from the final model, which contained variables recommended by multivariate least absolute shrinkage and selection operator Cox proportional hazards regression analysis. Their performance was quantified using the Harrell concordance index (C index), the time-dependent area under the receiver operating characteristic curve, and calibration curves and was compared with 6 prognostic systems. Recurrence-free survival was estimated by the Kaplan-Meier method, and RFS curves were compared using a log-rank test.

**Results:**

Among the 498 patients, 432 (86.7%) were men; the mean (SD) age at diagnosis was 51.4 (11.3) years. Independent predictors for RFS identified included VETC, MVI, tumor number, and maximum tumor size, which were incorporated into the multivariate model (VMNS model). The C index (0.702; 95% CI, 0.653-0.752) for the VMNS score of the training cohort was significantly higher than those of 6 conventional systems (0.587 [95% CI, 0.535-0.638] to 0.657 [95% CI, 0.606-0.708]). Different recurrence risk groups defined by the VMNS score showed significantly different 2-year RFS (low-risk group, 81.4% [SE, 0.036]; medium-risk group, 62.1% [SE, 0.054]; high-risk group, 30.1% [SE, 0.079]; *P* < .001). Calibration curves of the VMNS nomogram showed good agreement between the nomogram-predicted RFS probability and actual RFS proportion. The internal and external validation cohorts confirmed the results.

**Conclusions and Relevance:**

The VMNS model enabled individualized prognostication of RFS in patients with HCC undergoing curative resection.

## Key Points

**Question:**

Could integrating distinct vascular patterns with other crucial variables accurately predict recurrence risk in patients undergoing radical hepatectomy for hepatocellular carcinoma?

**Findings:**

In this multicenter prognostic cohort study of 498 patients undergoing radical hepatectomy, a multivariate model was developed and validated to reliably predict recurrence risk. A relative risk score exhibiting significantly better discrimination than conventional systems and dividing patients into significantly different prognostic groups and an absolute risk nomogram exhibiting satisfactory calibration were generated.

**Meaning:**

The findings suggest that by integrating distinct vascular patterns, the model-derived risk score and nomogram could enable individualized prognostication of recurrence risk for patients receiving radical hepatectomy.

## Teaser

This prognostic study establishes and validates a model based on vessels encapsulating tumor clusters and microvascular invasion to estimate recurrence-free survival in patients undergoing radical hepatectomy for hepatocellular carcinoma.

## Introduction

Hepatocellular carcinoma (HCC) is one of the most prevalent and lethal cancers worldwide.^1^ Among the potentially curative therapies, surgical resection is the primary option to improve the prognosis of patients.^2,3^ However, the long-term outcomes remain unsatisfactory owing to the high incidence of postoperative recurrence.^4^ The high heterogeneity of HCC makes it difficult to accurately identify the recurrence risk and develop appropriate interventions for individuals.

At present, predictors of recurrence-free survival (RFS) in HCC are inadequately defined, and the underlying mechanisms for metastasis and recurrence remain incompletely clarified. Epithelial-mesenchymal transition (EMT) has been considered a classic metastasis mechanism whereby single neoplastic cells undergo phenotype switching and obtain enhanced migration and invasion capacity.^5,6^ Epithelial-mesenchymal transition–mediated metastasis has been reported to promote the development of microvascular invasion (MVI),^7^ which is consistently identified as an indispensable predictor of RFS.^8,9^ In addition to MVI, several traditional clinical parameters such as multiple tumors have been recognized as significant predictors of RFS.^8,9,10^ However, in clinical practice, many patients without these key aggressive features still have recurrence after curative resection, indicating the insufficiency in depicting the heterogeneity and mechanism of HCC metastasis and recurrence.

Recently, Fang et al^11^ discovered a novel vascular pattern, vessels encapsulating tumor clusters (VETC), which was morphologically distinct from MVI and mediated a novel metastasis mechanism independent of EMT. Vessels encapsulating tumor clusters are endothelium-encapsulated tumor clusters that can be released into circulation directly through the anastomosis of VETC itself and peritumor vessels. Metastasis mediated by VETC is an important supplement to MVI for describing the heterogeneity of HCC recurrence. In addition, similar to MVI, VETC has been identified as a powerful pathological variable affecting survival in a large multinational study.^12^ The authors reported a 15.2% incidence of co-occurring VETC and MVI.^12^ Nevertheless, it remains unclear whether the integration of VETC and MVI can compensate for the insufficiency of the conventional clinicopathologic parameters in predicting HCC recurrence.

Thus, we conducted a large, multicenter cohort study with the aim of exploring and validating the prognostic value of integrating distinct vascular patterns in predicting HCC recurrence in patients undergoing curative resection. The recurrence risk score and nomogram generated were compared with other conventional staging systems.

## Methods

The results of our analysis are reported according to the Transparent Reporting of a Multivariable Prediction Model for Individual Prognosis or Diagnosis (TRIPOD) reporting guideline.^13^ This study was approved by the institutional ethical review boards of all the participating centers, and it was conducted in accordance with the ethical guidelines of the 1975 Declaration of Helsinki.^14^ Written informed consent was obtained from the patients for the use of their clinical data and formalin-fixed, paraffin-embedded tissue samples.

### Study Population

This multicenter, prognostic cohort study enrolled 498 patients from 5 independent hospitals in China: Sun Yat-Sen University Cancer Center, Guangzhou (n = 365); the First Affiliated Hospital of Guangzhou University of Chinese Medicine, Guangzhou (n = 47); Affiliated Cancer Hospital and Institute of Guangzhou Medical University, Guangzhou (n = 44); Fudan University Shanghai Cancer Center, Shanghai (n = 20); and Dongguan People’s Hospital, Dongguan City (n = 22). The patients who were treated at Sun Yat-Sen University Cancer Center from January 1, 2013, to December 31, 2014 (n = 365), were randomly assigned to the training cohort (243 [66.6%]) and internal validation cohort (122 [33.4%]) by computer-generated random numbers. The external validation cohort consisted of patients treated at the other 4 hospitals (n = 133) from January 1, 2013, to December 31, 2016.

The inclusion criteria for patients at all participating centers were as follows: (1) histologically confirmed primary HCC; (2) curative liver resection (R0 resection) as the initial treatment; (3) well-preserved liver function (Child-Pugh grade A or B); and (4) absence of macrovascular invasion (radiological evidence of major portal or hepatic vein invasion) and extrahepatic metastasis. The exclusion criteria consisted of (1) the presence of a second primary tumor; (2) incomplete clinical data or no follow-up data; and (3) no formalin-fixed, paraffin-embedded tissue samples available for immunohistochemical staining.

### Procedures

Data on demographics, laboratory tests, tumor pathology, tumor burden, and operative factors were derived from the patients’ medical records. Immunohistochemical analysis was performed to identify the pathological characteristics VETC and MVI. The HCC tissue samples were cut into 4-μm sections, dewaxed, and rehydrated. The sections underwent quenching of endogenous peroxidase activity in 0.3% hydrogen peroxide, high-pressure antigen retrieval in 10 mM citrate buffer (pH, 6.0), and incubation with primary antibody at 4 °C overnight. Staining was performed using a commercially available detection system (EnVision; Dako Cytomation). Sections were counterstained with hematoxylin-eosin. Pathological reports of surgical specimens were used as the reference standard for VETC and MVI, which were analyzed by 2 experienced pathologists (J.-C.F. and Y.-H.L.) by consensus. Vessels encapsulating tumor clusters refers to sinusoidal blood vessels (CD34^+^) encapsulating individual tumor clusters and forming cobweblike networks.^11^ The area of VETC was assessed semiquantitatively in 5% of the units. For the selection of cutoff value for VETC, 2 previous single-center studies^11,15^ defined the presence of the VETC pattern as VETC positivity and the absence of the VETC pattern as VETC negativity, whereas another multinational study with a large sample size^12^ set 55% as the optimal cutoff value using the K-adaptive partitioning algorithm. No consensus on the choice of a detection threshold for VETC has been reached. Considering that varied and changing pathological criteria would result in confusion in the clinical application, we adopted the cutoff value (55%) from the latter study but did not redefine a cutoff value. That is, the appearance of VETC in 55% or more of the tumor surface (ranging from 0%-100%) was defined as VETC positivity. Microvascular invasion positivity was defined as microscopic appearance of neoplastic emboli within the intratumoral vessel lumen or neoplastic cells invading the vessel wall.^16,17^

We staged all patients according to 6 universally used prognostic systems: the Barcelona Clinic Liver Cancer staging system,^18^ the 8th edition of the American Joint Committee on Cancer tumor-node-metastasis (TNM) staging system,^19^ the Hong Kong Liver Cancer staging system,^20^ the Japan Integrated Staging score,^21^ the Tokyo score,^22^ and the Chinese University Prognostic Index score.^23^ After curative resection, the patients were followed up with dynamic enhanced computed tomography, magnetic resonance imaging, ultrasonography, measurement of serum α-fetoprotein levels, and other laboratory tests related to liver function. The postoperative follow-up evaluation was performed 1 month after resection, every 3 months in the first 2 years, and every 3 to 6 months subsequently. The end point of RFS was defined as the interval from the date of curative hepatic resection to the date of recurrence with a new lesion confirmed by at least 2 radiological imaging modalities or the last known follow-up. Patients alive without evidence of recurrence at last follow-up were censored. Patients were followed up until March 30, 2020.

### Statistical Analysis

The data were analyzed from December 1 to 31, 2020. Continuous variables with a normal distribution are expressed as the mean (SD), and those with a nonnormal distribution are expressed as the median (interquartile range [IQR]). Categorical variables are expressed as numbers and percentages. The maximum tumor size and α-fetoprotein level were logarithmically transformed for the univariate and multivariate Cox proportional hazards regression analyses owing to their original approximately log-normal (ln) distribution. The correlation between VETC and MVI was analyzed using the Pearson χ^2^ test. The prognostic value of VETC and 22 other clinicopathological parameters for RFS was preliminarily explored by univariate Cox proportional hazards regression analyses in the training cohort. Given that the bivariable selection method was inappropriate for selecting variables, no further variable screening was implemented out of concern about wrongly rejecting potentially important variables whose correlation with RFS was masked by confounders.^24^ That is, all 23 variables were incorporated into the multivariate regularized Cox proportional hazards regression with the least absolute shrinkage and selection operator (LASSO), which outperformed stepwise in the field of variable selection.^25^ LASSO Cox proportional hazards regression was implemented using the glmnet R package.^26^ This multivariate modeling approach shrinks each coefficient estimate and sets some of them toward zero to eliminate nonvaluable predictors. The parameter λ determines the degree of shrinkage and the variables used to refit a Cox proportional hazards regression model. The Cox proportional hazards regression model was built based on the training cohort and applied to the validation cohorts. Corresponding to different optimal values of λ ranging from minimum criteria to 1–standard error criteria, different ideal schemes of variable selection were attempted, and we compared the performance of the different Cox proportional hazards regression models by comparing C statistics and the likelihood ratio test in all the cohorts to determine the best model. Furthermore, we compared the performances of the nested models with and without some important variables (eg, MVI, VETC) by comparing C statistics and the likelihood ratio test in the 3 cohorts.

In terms of the relative risk of recurrence, the formula of the risk score was the linear component of the exponential function in the multivariate Cox proportional hazards regression model. The relative risk score consisted of the weighted sum of the selected predictors, and the weights were the β estimates of the predictors in the multivariate Cox proportional hazards regression model. The discriminatory ability of the relative risk score was assessed by the Harrell concordance index (C index) and the time-dependent area under the receiver operating characteristic curve (tdAUROC), which were compared with those of other prognostic systems (Barcelona Clinic Liver Cancer stage, TNM stage, Hong Kong Liver Cancer stage, Japan Integrated Staging score, Tokyo score, and Chinese University Prognostic Index score). The C indices of the models were compared using the compareC R package through the approach proposed by Kang et al.^27^ The tdAUROCs of the models were compared using the timeROC R package.^28^ Patients were also stratified into 3 risk groups (low, medium and high) by the 50th and 85th percentiles of the relative risk score in the training cohort based on a previously reported statistical approach for the validation of the Cox proportional hazards regression model.^29^ The RFS curves of the different risk groups were plotted using the Kaplan-Meier method, and the curves were compared using a log-rank test. The relative risk score and risk group for an individual were also applied to the internal and external validation cohorts. Given that the predicted absolute RFS probability is of more clinical significance, a nomogram was constructed based on the final multivariate model. Calibration curves were plotted to compare the nomogram-predicted RFS probability and actual RFS proportion. In a model performing well in calibration, the points are close to the ideal 45-degree line.

We performed bootstrapping with 1000 resamples to calculate the C index and tdAUROC and to construct the calibration curves. All statistical analyses were performed in R, version 3.6.1 (R Program for Statistical Computing). All statistical tests were 2 sided. *P* < .05 was considered statistically significant.

## Results

### Baseline Characteristics of the Patients

The 498 patients included in the analysis consisted of 432 men (86.7%) and 66 women (13.3%). Men constituted 208 of 243 patients (85.6%) in the training cohort, 111 of 122 (91.0%) in the internal validation cohort, and 113 of 133 (85.0%) in the external validation cohort. The mean (SD) age at diagnosis was 51.4 (11.3) years overall, 50.8 (11.5) years in the training cohort, 50.2 (10.6) years in the internal validation cohort, and 53.7 (11.4) years in the external validation cohort (eTable 1 in the Supplement). Most patients had hepatitis B virus infection (214 [88.1%] in the training cohort, 108 [88.5%] in the internal validation cohort, and 113 [85.0%] in the external validation cohort). The median maximum tumor size was 4.8 (IQR, 3.1-7.0) cm in the training cohort, 4.5 (IQR, 3.2-6.4) cm in the internal validation cohort, and 5.0 (IQR, 3.5-7.0) cm in the external validation cohort. Most patients had a single tumor (211 [86.8%] in the training cohort, 106 [86.9%] in the internal validation cohort, and 105 [78.9%] in the external validation cohort). The proportions of patients with a VETC-positive phenotype (morphological features shown in eFigure 1 in the Supplement) were 64 (26.3%) in the training cohort, 23 (18.9%) in the internal validation cohort, and 24 (18.0%) in the external validation cohort. Microvascular invasion was observed via microscope in 91 patients (37.4%) in the training cohort, 42 (34.4%) in the internal validation cohort, and 35 (26.3%) in the external validation cohort. The incidences of co-occurrence of VETC and MVI were 36 patients (14.8%) in the training cohort, 11 (9.0%) in the internal validation cohort, and 11 (8.3%) in the external validation cohort. Patients with a positive VETC phenotype were more likely to have a positive MVI phenotype in the entire cohort (VETC and MVI negativity, 277 [55.6%]; VETC negativity and MVI positivity, 110 [22.1%]; VETC positivity and MVI negativity, 53 [10.6%]; and VETC and MVI positivity, 58 [11.6%]; *P* < .001). The median follow-up times were 60.5 (IQR, 52.4-67.8) months in the training cohort, 65.5 (IQR, 57.5-71.5) months in the internal validation cohort, and 52.9 (IQR, 37.9-58.4) months in the external validation cohort. Recurrence was recorded in 98 patients (40.3%) in the training cohort, 69 (56.6%) in the internal validation cohort, and 70 (52.6%) in the external validation cohort. The mean (SD) times to recurrence were 51.8 (2.2) months in the training cohort, 41.4 (3.1) months in the internal validation cohort, and 44.0 (3.2) months in the external validation cohort. The mean (SD) event-free survival (without recurrence or death from any cause) was 48.9 (2.2) months in the training cohort, 39.8 (3.1) months in the internal validation cohort, and 39.3 (3.0) months in the external validation cohort.

### Construction of the Multivariate Cox Proportional Hazards Regression Model

The results of univariate Cox proportional hazards regression analyses of prognostic factors for RFS in the training cohort are reported in Table 1. The process of variable selection using the multivariate LASSO Cox proportional hazards regression model is shown in eFigure 2 in the Supplement. Corresponding to different optimal values of λ ranging from minimum criteria to 1-SE criteria, 2 ideal schemes of variable selection were recommended. The first scheme contains 4 variables (VETC, MVI, tumor number, and maximum tumor size [ln]), whereas the other contains 5 variables (the 4 variables plus tumor differentiation). The model containing 4 variables was attempted first, with hazard ratios (HRs) of 1.85 (95% CI, 1.20-2.86; *P* = .005) for VETC, 1.78 (95% CI, 1.15-2.77; *P* = .01) for MVI, 2.54 (95% CI, 1.53-4.20; *P* < .001) for tumor number, and 1.71 (95% CI, 1.17-2.51; *P* = .006) for maximum tumor size (ln) (Table 1). When adding the variable tumor differentiation, the C indices were slightly higher than those of the multivariable VETC-MVI-number-size (VMNS) model without statistical significance (0.712 [95% CI, 0.665-0.760] for the training cohort; 0.675 [95% CI, 0.612-0.737] for the internal validation cohort; and 0.730 [95% CI, 0.676-0.784] for the external validation cohort; *P* > .05 for all) (eTable 2 in the Supplement). However, the C index is insensitive to the significant improvements of an additional predictor to the basic model because it only takes into account the discrimination ability between the models.^30^ The likelihood ratio test was recommended to test the differences between models with and without a specific variable in both the calibration and discrimination abilities.^30^ Because no significant improvements were obtained in the likelihood ratio test by adding tumor differentiation to the VMNS model (χ^2^ = 2.574 for the training cohort; χ^2^ = 0.074 for the internal validation cohort; χ^2^ = 1.357 for the external validation cohort; *P* > .05 for all) (eTable 2 in the Supplement), we finally chose the VMNS model to establish the score for relative recurrence risk and the nomogram for absolute RFS probability.

**Table 1.** Univariate and Multivariate Cox Proportional Hazards Regression Analyses of Prognostic Factors for RFS in the Training Cohort

Variable	Univariate analysis		Multivariate analysis		
	HR (95% CI)	*P* value	HR (95% CI)	β estimate	*P* value
Age (>50 vs ≤50 y)	0.751 (0.502-1.124)	.16	NA	NA	NA
Sex (male vs female)	1.747 (0.879-3.470)	.11	NA	NA	NA
HBV infection (positive vs negative)	1.261 (0.655-2.428)	.49	NA	NA	NA
HCV infection (positive vs negative)	2.141 (0.298-15.380)	.45	NA	NA	NA
Platelet count (>100 × 10^3^/μL vs ≤100 × 10^3^/μL)	1.200 (0.623-2.310)	.59	NA	NA	NA
Albumin level (>3.5 vs ≤3.5 g/dL)	2.276 (0.924-5.606)	.07	NA	NA	NA
Total bilirubin level (>1.0 vs ≤1.0 mg/dL)	0.966 (0.590-1.581)	.89	NA	NA	NA
AFP level in ng/mL (ln)	1.071 (1.007-1.139)	.03	NA	NA	NA
Prothrombin time (>13.5 vs ≤13.5 s)	1.190 (0.484-2.929)	.71	NA	NA	NA
Neutrophil count (>6300/μL vs ≤6300/μL)	1.442 (0.769-2.705)	.25	NA	NA	NA
WBC count (>10 × 10^3^/μL vs ≤10 × 10^3^/μL)	0.965 (0.392-2.376)	.94	NA	NA	NA
CRP level (>0.3 vs ≤0.3 mg/dL)	1.661 (1.108-2.491)	.01	NA	NA	NA
ALT level (>40 vs ≤40 U/L)	1.289 (0.864-1.925)	.21	NA	NA	NA
AST level (>35 vs ≤35 U/L)	1.420 (0.949-2.127)	.09	NA	NA	NA
Liver cirrhosis (present vs absent)	1.200 (0.794-1.814)	.39	NA	NA	NA
Child-Pugh grade (B vs A)	1.614 (0.225-11.602)	.63	NA	NA	NA
VETC (positive vs negative)	2.349 (1.554-3.553)	<.001	1.853 (1.203-2.855)	0.617	.005
MVI (positive vs negative)	2.628 (1.756-3.933)	<.001	1.780 (1.146-2.766)	0.577	.01
No. of tumors (multiple vs single)	2.666 (1.626-4.371)	<.001	2.537 (1.533-4.200)	0.931	<.001
Maximum tumor size in cm (ln)	2.266 (1.561-3.290)	<.001	1.714 (1.170-2.511)	0.539	.006
Tumor differentiation (poor/moderate vs well)	2.615 (1.063-6.436)	.04	NA	NA	NA
Anatomical resection (yes vs no)	1.606 (1.050-2.458)	.03	NA	NA	NA
Surgical margin (>1 vs ≤1 cm)	0.822 (0.539-1.252)	.36	NA	NA	NA

Abbreviations: AFP, α-fetoprotein; ALT, alanine aminotransferase; AST, aspartate aminotransferase; CRP, C-reactive protein; HBV, hepatitis B virus; HCV, hepatitis C virus; HR, hazard ratio; ln, log normal; MVI, microvascular invasion; NA, not applicable; RFS, recurrence-free survival; VETC, vessels encapsulating tumor clusters; WBC, white blood cell.

SI conversion factors: To convert albumin to g/L, multiply by 10; ALT and AST to μkat/L, multiply by 0.0167; bilirubin to μmol/L, multiply by 17.104; CRP to mg/L, multiply by 10; neutrophil count and WBC to ×10^9^/L, multiply by 0.001; platelets to ×10^9^/L, multiply by 1.0.

We also tried to delete VETC or MVI from the VMNS model to explore the contributions of VETC and MVI to the prediction of HCC recurrence. The results of the comparison between the nested models are reported in eTable 2 in the Supplement. The C indices of the model with VETC were 0.702 (95% CI, 0.653-0.752) in the training cohort, 0.673 (95% CI, 0.611-0.735) in the internal validation cohort, and 0.720 (95% CI, 0.665-0.776) in the external validation cohort. The C indices of the model without VETC were 0.696 (95% CI, 0.646-0.745) in the training cohort, 0.643 (95% CI, 0.579-0.707) in the internal validation cohort, and 0.704 (95% CI, 0.648-0.759) in the external validation cohort. No significant differences in the C index were found between the nested models with and without VETC in the training cohort (0.702 [95% CI, 0.653-0.752] and 0.696 [95% CI, 0.646-0.745], respectively) and in the external validation cohort (0.720 [95% CI, 0.665-0.776] and 0.704 [95% CI, 0.648-0.759], respectively; *P* > .05 for all). However, the likelihood ratio test demonstrated a significant influence of deleting VETC from the VMNS model (χ^2^ = 7.453 [*P* = .006] in the training cohort; χ^2^ = 8.190 [*P* = .004] in the internal validation cohort; χ^2^ = 5.771 [*P* = .02] in the external validation cohort). Similar findings were obtained when deleting MVI or even deleting both VETC and MVI from the VMNS model (eTable 2 in the Supplement). Given that the likelihood ratio test is superior to the C index when comparing models with and without a specific variable,^30^ all aforementioned results confirm the nonnegligible role of VETC in predicting HCC recurrence, which is no less important than MVI. In addition, in the entire cohort, the 2-year RFS rates were 21.4% (SE, 0.056) in the group with VETC and MVI positivity, 49.6% (SE, 0.070) in the group with VETC positivity and MVI negativity, 51.3% (SE, 0.049) in the group with VETC negativity and MVI positivity, and 74.9% (SE, 0.027) in the group with VETC and MVI negativity. The 5-year RFS rates were 14.8% (SE, 0.050) in the group with VETC and MVI positivity, 42.1% (SE, 0.072) in the group with VETC positivity and MVI negativity, 43.0% (SE, 0.050) in the group with VETC negativity and MVI positivity, and 62.3% (SE, 0.031) in the group with VETC and MVI negativity.

### VMNS Score for Relative Recurrence Risk

The relative risk score based on the final VMNS model was calculated as follows: VMNS score = 0.617 × VETC (0, negative; 1, positive) + 0.577 × MVI (0, negative; 1, positive) + 0.931 × tumor number (0, single; 1, multiple) + 0.539 × maximum tumor size in cm (ln). The VMNS score was obtained for each individual in each cohort. In the training cohort, the VMNS score had a significantly higher C index (0.702; 95% CI, 0.653-0.752) than those of the other 6 staging systems (0.587 [95% CI, 0.535-0.638] to 0.657 [95% CI, 0.606-0.708]) (Table 2). For the validation cohorts, the VMNS scores also had significantly higher C index values (internal validation cohort, 0.673 [95% CI, 0.611-0.735] vs 0.522 [95% CI, 0.456-0.588] to 0.607 [95% CI, 0.547-0.667]; external validation cohort, 0.720 [95% CI, 0.665-0.776] vs 0.577 [95% CI, 0.526-0.628] to 0.646 [95% CI, 0.591-0.701]) (Table 2).

**Table 2.** C Index of Different Models of Recurrence-Free Survival in the Training and Validation Cohorts

Prognostic model	Training cohort		Internal validation cohort		External validation cohort	
	C index (95% CI)	*P* value^a^	C index (95% CI)	*P* value^a^	C index (95% CI)	*P* value^a^
VMNS score	0.702 (0.653-0.752)	NA	0.673 (0.611-0.735)	NA	0.720 (0.665-0.776)	NA
TNM stage	0.646 (0.594-0.698)	.004	0.605 (0.543-0.666)	.008	0.637 (0.580-0.695)	.001
BCLC stage	0.587 (0.546-0.628)	<.001	0.555 (0.505-0.606)	<.001	0.577 (0.526-0.628)	<.001
HKLC stage	0.631 (0.582-0.680)	.004	0.607 (0.547-0.667)	.008	0.637 (0.586-0.689)	.002
JIS score	0.657 (0.606-0.708)	.008	0.604 (0.542-0.666)	.003	0.646 (0.591-0.701)	.001
Tokyo score	0.619 (0.569-0.669)	.001	0.592 (0.529-0.655)	.004	0.623 (0.565-0.681)	.001
CUPI score	0.587 (0.535-0.638)	<.001	0.522 (0.456-0.588)	<.001	0.591 (0.535-0.648)	<.001

Abbreviations: BCLC, Barcelona Clinic Liver Cancer; C index, Harrell concordance index; CUPI, Chinese University Prognostic Index; HKLC, Hong Kong Liver Cancer; JIS, Japan Integrated Staging; NA, not applicable; TNM, 8th edition of the American Joint Committee on Cancer tumor-node-metastasis staging system; VMNS, vessels encapsulating tumor clusters–microvascular invasion-number-size.

^a^ Compared with VMNS score.

**Figure 1.**
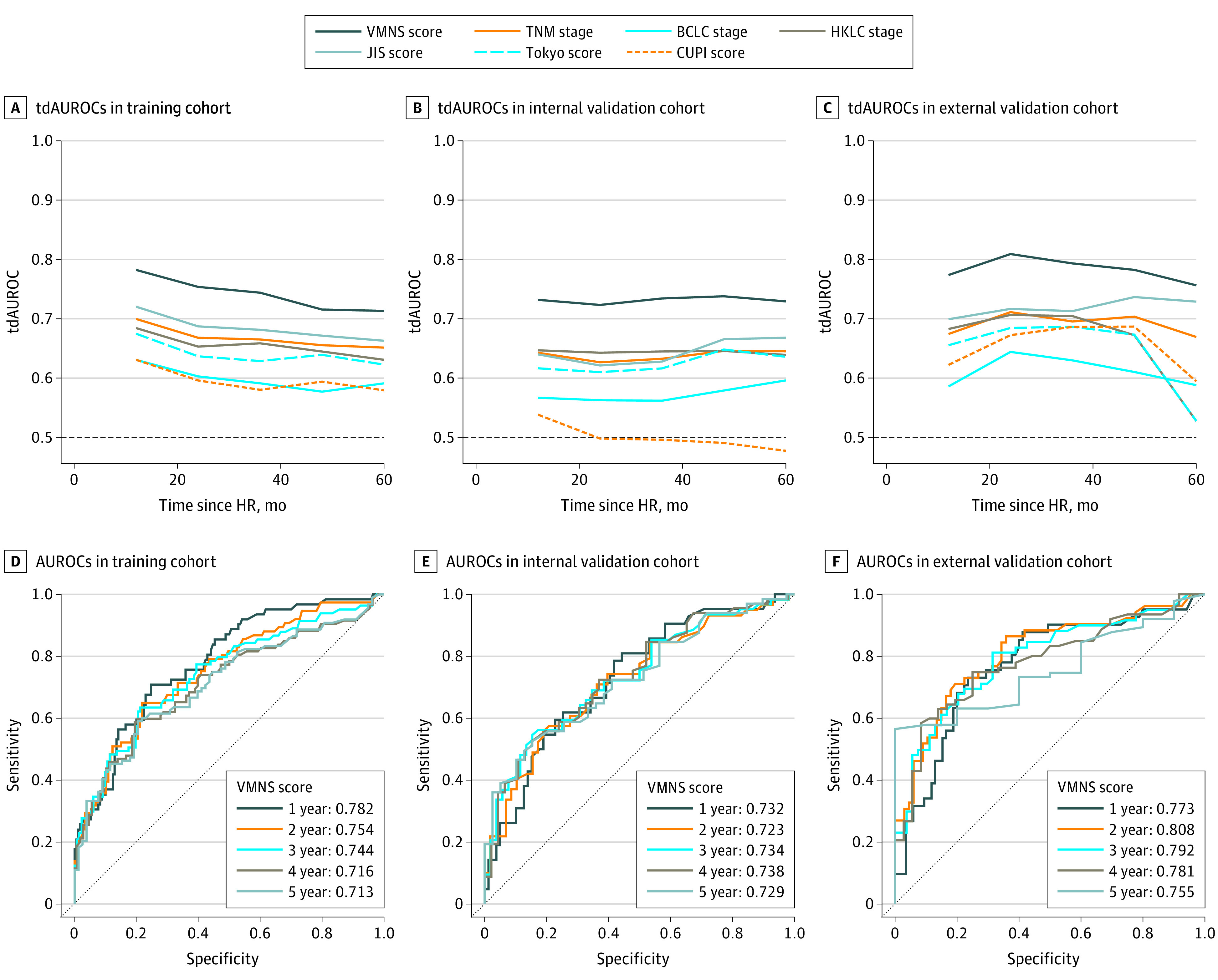
Time-Dependent Area Under the Receiver Operating Characteristic Curve (tdAUROC) Value Over Time of the 7 Prognostic Systems Since Hepatic Resection (HR) and Exact AUROCs of the Vessels Encapsulating Tumor Clusters–Microvascular Invasion–Number-Size (VMNS) Score in Each Cohort BCLC indicates Barcelona Clinic Liver Cancer; CUPI, Chinese University Prognostic Index; HKLC, Hong Kong Liver Cancer; JIS, Japan Integrated Staging; TNM, 8th edition of the American Joint Committee on Cancer tumor-node-metastasis staging system; and VMNS, vessels encapsulating tumor clusters–microvascular invasion–number–size.

The tdAUROC of the VMNS score was always higher than those of the other 6 systems in each cohort over time (Figure 1A-C). For instance, the 2-year tdAUROC of the VMNS score was significantly higher than those of the other staging systems (training cohort, 0.754 [95% CI, 0.686-0.822] vs 0.596 [95% CI, 0.528-0.664] to 0.687 [95% CI, 0.619-0.755]; internal validation cohort, 0.723 [95% CI, 0.631-0.815] vs 0.498 [95% CI, 0.406-0.590] to 0.643 [95% CI, 0.554-0.732]; external validation cohort, 0.808 [95% CI, 0.727-0.889] vs 0.644 [95% CI, 0.569-0.718] to 0.716 [95% CI, 0.633-0.799]) (eTable 3 in the Supplement). For the training cohort, the 1-year AUROC of the VMNS score was 0.782 (95% CI, 0.717-0.848); the 2-year AUROC, 0.754 (95% CI, 0.686-0.822); the 3-year AUROC, 0.744 (95% CI, 0.675-0.813); the 4-year AUROC, 0.716 (95% CI, 0.641-0.790); and the 5-year AUROC, 0.713 (95% CI, 0.636-0.790) (Figure 1D). For the internal validation cohort, the 1-year AUROC of the VMNS score was 0.732 (95% CI, 0.639-0.825); the 2-year AUROC, 0.723 (95% CI, 0.631-0.815); the 3-year AUROC, 0.734 (95% CI, 0.643-0.825); the 4-year AUROC, 0.738 (95% CI, 0.646-0.829); and the 5-year AUROC, 0.729 (95% CI, 0.634-0.824) (Figure 1E). For the external validation cohort, the 1-year AUROC of the VMNS score was 0.773 (95% CI, 0.682-0.863); the 2-year AUROC, 0.808 (95% CI, 0.727-0.889); the 3-year AUROC, 0.792 (95% CI, 0.709-0.875); the 4-year AUROC, 0.781 (95% CI, 0.690-0.873); and the 5-year AUROC, 0.755 (95% CI, 0.636-0.874) (Figure 1F).

**Figure 2.**
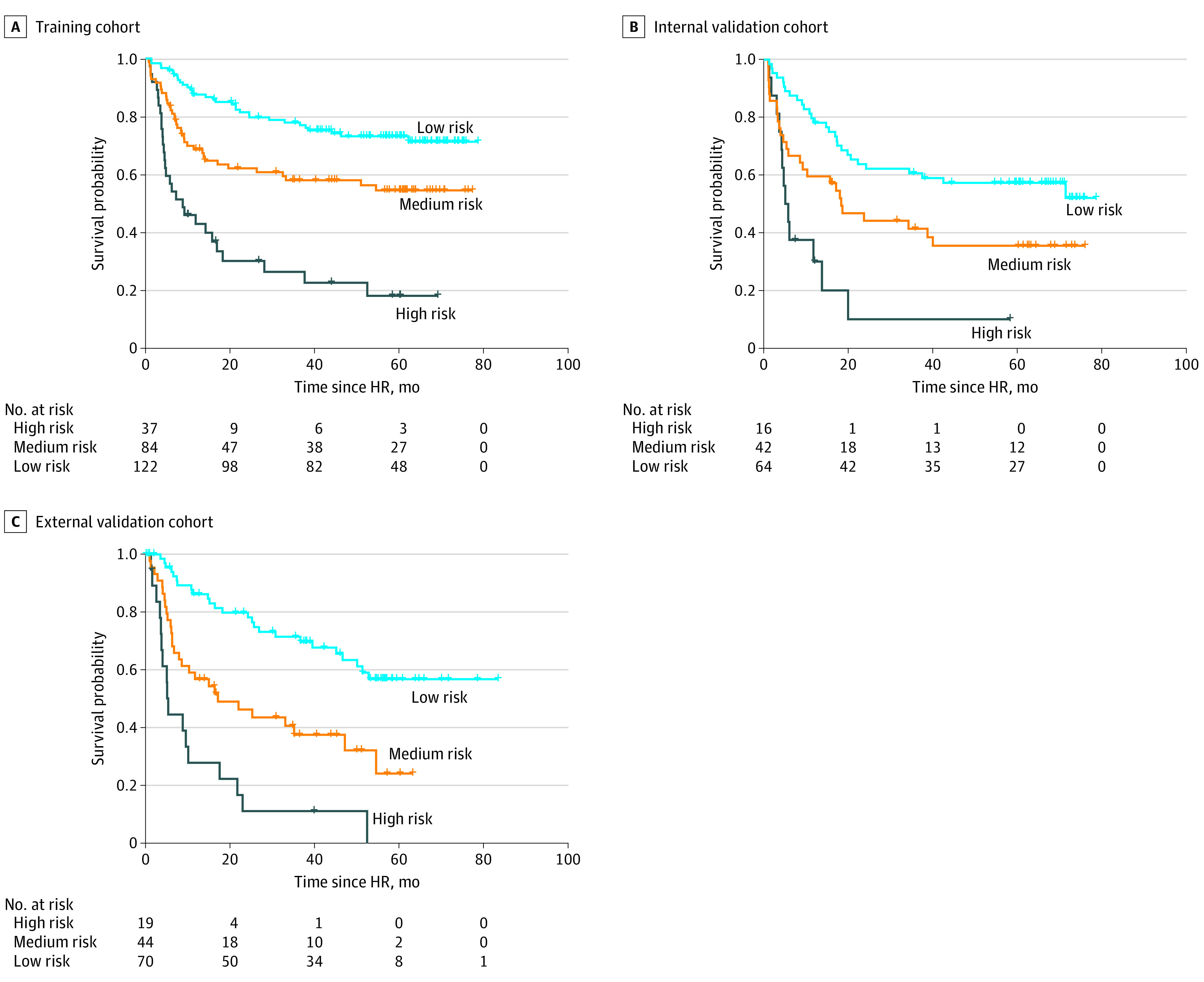
Kaplan-Meier Recurrence-Free Survival Curves of Different Risk Groups in Each Cohort for the Entire Follow-up Period Since Curative Hepatic Resection (HR) *P* < .001 between risk groups. Risk groups were derived from the proposed vessels encapsulating tumor clusters–microvascular invasion–number–size score.

Using −0.148 and 0.765 (which corresponded to the 50th and 85th percentiles of the VMNS score in the training cohort) as the cutoff values, the patients from each cohort were stratified into 3 distinct recurrence risk groups (low, medium, and high). The RFS curves for the different risk groups were significantly different (*P* < .001 in each cohort, Figure 2). For the training cohort, the 2- and 5-year RFS rates were 81.4% (SE, 0.036) and 73.1% (SE, 0.042), respectively, in the low-risk group; 62.1% (SE, 0.054) and 54.5% (SE, 0.057), respectively, in the medium-risk group (HR, 2.05; 95% CI, 1.25-3.37; *P* < .001); and 30.1% (SE, 0.079) and 18.0% (SE, 0.072), respectively, in the high-risk group (HR, 5.47; 95% CI, 2.62-11.38; *P* < .001) (eTable 4 in the Supplement). Similar conclusions were drawn for the internal and external validation cohorts (eTable 4 in the Supplement).

### VMNS Nomogram for Absolute RFS Probability

**Figure 3.**
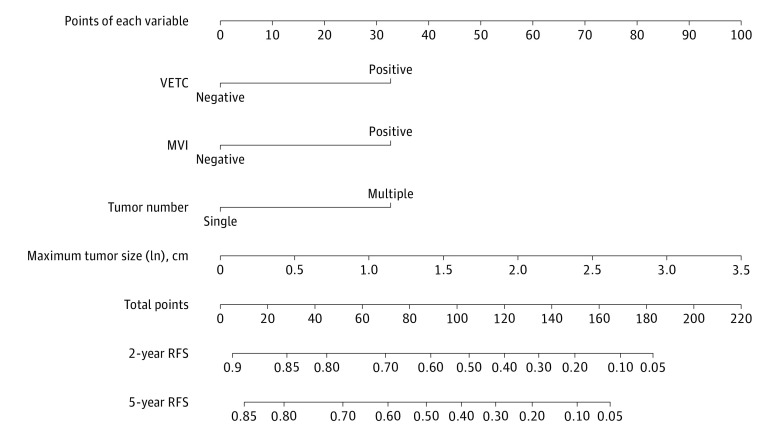
Nomogram for Recurrence-Free Survival (RFS) of Patients With Hepatocellular Carcinoma Undergoing Curative Resection To use the nomogram, locate an individual patient’s value on each independent variable axis, and then draw a line upward to obtain the points for each variable. Next, locate the sum of these points on the total points axis, and draw a line downward to the recurrence axis to obtain the probability of 2- or 5-year RFS. MVI indicates microvascular invasion; VETC, vessels encapsulating tumor clusters.

Using the coefficients of the VMNS model, the VMNS nomogram (Figure 3) was built to predict the absolute RFS probability for each individual. The absolute RFS probability refers to the probability of survival without recurrence. The calibration curves of the VMNS nomogram showed good agreement between the nomogram-predicted RFS probability and actual RFS proportion in each cohort (eFigure 3 in the Supplement). The dynamic VMNS nomogram for predicting the RFS probability is available online.^31^ The dynamic VMNS nomogram is more accurate, convenient, and flexible for recurrence prediction in clinical applications. By simply choosing or entering the characteristics of the 4 variables (VETC: positive or negative; MVI: positive or negative; tumor number: multiple or single; maximum tumor size in cm [ln]) on the web page, clinicians can obtain the absolute RFS probability at different times after curative hepatic resection for each patient.

## Discussion

In this large multicenter cohort study, by integrating distinct vascular patterns, we developed and validated a VMNS model for recurrence prediction in patients with HCC undergoing curative hepatectomy. In terms of tools for relative recurrence risk, compared with common prognostic systems, the VMNS score displayed superior prognostic performance consistently during follow-up. By calculating the VMNS score for an individual, clinicians can stratify patients into prognostically distinct risk groups and recommend closer clinical follow-up for high-risk patients. Regarding the absolute RFS probability, the dynamic VMNS nomogram further provides a practical and well-calibrated risk predictive tool for future clinical application.

The high recurrence rate of HCC has hampered the long-term survival of patients undergoing curative resection,^4^ and the prediction of HCC recurrence remains inadequate. A few key variables (such as MVI, large tumor size, and multiple tumors) have been studied adequately and identified as significant predictors of RFS.^8,9,10^ However, tumor relapse remains frequent even in patients without these aggressive features. In addition, integrating these clinical variables seemed insufficient to delineate the heterogeneity of HCC and predict HCC recurrence. Alternative models have been proposed based on genomic predictors,^32,33,34^ but they have not been widely used due to the complex molecular basis and technologies.^35,36^ On the other hand, little attention has been paid to another aggressive pathologic feature, VETC, which is significantly associated with a worse RFS^12^ but morphologically and functionally different from MVI.^11^ To our knowledge, the present study is the first to underscore the importance of integrating these 2 distinct vascular patterns for recurrence prediction. In our results, VETC was no less important than MVI in predicting HCC recurrence (with a slightly higher weight than MVI in the VMNS model). Patients with both a positive phenotype of VETC and MVI have a higher recurrence risk than those with only 1 positive phenotype, whereas patients with only VETC positivity or only MVI positivity have a similar recurrence risk. Vessels encapsulating tumor clusters and MVI are positively correlated with each other, and the underlying mechanism remains unclear. The additional use of VETC may help compensate for the insufficiency in describing tumor heterogeneity and better predict HCC recurrence. Moreover, the clinical detection of VETC is convenient, inexpensive, and easily applicable.

Early metastasis is a leading cause of HCC recurrence.^37^ However, the underlying mechanisms of HCC metastasis remain incompletely elucidated. Epithelial-mesenchymal transition has been considered the classic metastasis mechanism, whereby epithelial cells change into a mesenchymal phenotype temporarily and acquire an enhanced capacity to spread to surrounding and distant tissues.^5,6^ Epithelial-mesenchymal transition has been reported to promote the development of MVI.^7^ Furthermore, individual circulating neoplastic cells within microvessels exhibit mesenchymal morphology according to microscopic examination. Nevertheless, accumulating evidence suggests that the number of circulating tumor clusters is more robustly associated with tumor metastasis and relapse than the number of individual circulating tumor cells.^15,38^ Fang et al^11^ revealed that a novel vascular pattern, VETC, promoted the release of endothelium-encapsulated tumor clusters into blood circulation directly through the anastomosis of VETC itself with peritumor vessels. More crucially, the metastasis mechanism mediated by VETC is independent of EMT, which indicates that VETC is a good complement to metastatic heterogeneity. Compared with single circulating neoplastic cells via EMT-mediated metastasis, circulating endothelium-encapsulated tumor clusters via VETC-mediated metastasis are protected from anoikis and immune assaults. Thus, investigators have proposed that VETC-mediated metastasis is more efficient than EMT-mediated metastasis.^11^ Therefore, VETC may be complementary to MVI in the predictive value for recurrence, as our results show. The integration of 2 distinct vascular patterns, VETC and MVI, may provide a more comprehensive view of the nature of tumor metastasis and recurrence and further allow better prediction of tumor relapse when considered together with other key variables.

### Limitations

The present study has several limitations. First, it is a retrospective cohort study, and thus, deviation may be unavoidable. To address this limitation, we conducted a large, multicenter study. Second, this study was conducted in an area where the hepatitis B virus is endemic. The validation of the VMNS model in other populations with different causes of HCC remain to be explored. Third, no consensus about the optimal cutoff to determine the VETC phenotype has been reached. Thus, a large prospective study will be necessary to determine the optimal threshold. In addition, the 2- and 5-year RFS rates according to the risk scores were different in the 3 cohorts, which remains to be explored by a prospective study with a larger sample size.

## Conclusions

In this prognostic cohort study, 2 distinct vascular patterns, VETC and MVI, were integrated into the VMNS model for predicting HCC recurrence after curative resection. Based on the VMNS model, the VMNS score and dynamic VMNS nomogram display excellent discrimination and calibration abilities, showing great potential in future clinical application to help guide surveillance and improve the long-term survival outcomes of patients.

## Notes

Supplement. **eTable 1.** Baseline Characteristics of the Patients in the Training and Validation Cohorts **eTable 2.** Contributions of Different Variables to the Prediction of HCC Recurrence **eTable 3.** 2-Year AUROC of Different Models of Recurrence-Free Survival in the Training and Validation Cohorts **eTable 4.** Hazard Ratio and 2- and 5-Year Recurrence-Free Survival According to Each Risk Group Defined by the VMNS Score **eFigure 1.** Representative Morphological Features of VETC in HCC Tissues by Immunohistochemical Staining of Human CD34 **eFigure 2.** Variable Selection Using LASSO Cox Proportional Hazards Regression Model **eFigure 3.** Calibration Curves of the VMNS Nomogram at 2 Years in Each Cohort
