# Supplementary material for: Development and Validation of a Model Including Distinct Vascular Patterns to Estimate Survival in Hepatocellular Carcinoma
Source: JAMA Netw Open. 2021 Sep 13;4(9):e2125055. doi: 10.1001/jamanetworkopen.2021.25055 (PMC8438596; doi:10.1001/jamanetworkopen.2021.25055)
Supplement: Supplement. — eTable 1. Baseline Characteristics of the Patients in the Training and Validation Cohorts eTable 2. Contributions of Different Variables to the Prediction of HCC Recurrence eTable 3. 2-Year AUROC of Different Models of Recurrence-Free Survival in the Training and Validation Cohorts eTable 4. Hazard Ratio and 2- and 5-Year Recurrence-Free Survival According to Each Risk Group Defined by the VMNS Score eFigure 1. Representative Morphological Features of VETC in HCC Tissues by Immunohistochemical Staining of Human CD34 eFigure 2. Variable Selection Using LASSO Cox Proportional Hazards Regression Model eFigure 3. Calibration Curves of the VMNS Nomogram at 2 Years in Each Cohort [file jamanetwopen-e2125055-s001.pdf]

Supplementary Online Content

Lin WP, Xing KL, Fu JC, et al. Development and validation of a model including distinct vascular patterns to estimate survival in hepatocellular carcinoma. *JAMA Netw Open*. 2021;4(9):e2125055. doi:10.1001/jamanetworkopen.2021.25055

eTable 1. Baseline Characteristics of the Patients in the Training and Validation Cohorts
eTable 2. Contributions of Different Variables to the Prediction of HCC Recurrence
eTable 3. 2-Year AUROC of Different Models of Recurrence-Free Survival in the Training and Validation Cohorts
eTable 4. Hazard Ratio and 2- and 5-Year Recurrence-Free Survival According to Each Risk Group Defined by the VMNS Score
eFigure 1. Representative Morphological Features of VETC in HCC Tissues by Immunohistochemical Staining of Human CD34
eFigure 2. Variable Selection Using LASSO Cox Proportional Hazards Regression Model
eFigure 3. Calibration Curves of the VMNS Nomogram at 2 Years in Each Cohort

This supplementary material has been provided by the authors to give readers additional information about their work.

© 2021 Lin WP et al. *JAMA Network Open*.

eTable 1. Baseline Characteristics of the Patients in the Training and Validation Cohorts

Variable	Training cohort (n = 243)	Internal validation cohort (n = 122)	External validation cohort (n = 133)
Age, years	50.8 ± 11.5	50.2 ± 10.6	53.7 ± 11.4
Sex			
female	35 (14.4)	11 (9.0)	20 (15.0)
male	208 (85.6)	111 (91.0)	113 (85.0)
HBV infection			
absent	29 (11.9)	14 (11.5)	20 (15.0)
present	214 (88.1)	108 (88.5)	113 (85.0)
HCV infection			
absent	242 (99.6)	119 (97.5)	128 (96.2)
present	1 (0.4)	3 (2.5)	5 (3.8)
PLT, 10^9/L	173.0 (136.5, 214.2)	169.9 (131.8, 200.8)	168.0 (115.0, 229.0)
ALB, g/L	43.0 (40.7, 44.8)	42.6 (40.5, 45.3)	42.1 (38.0, 45.0)
Total bilirubin, μmol/L	12.7 (10.2, 16.4)	13.1 (10.2, 16.2)	14.4 (11.1, 21.8)
AFP, ng/ml	50.3 (5.7, 873.8)	128.7 (6.4, 1574.8)	41.8 (6.7, 422.1)
PT, sec	11.7 (11.1, 12.3)	11.7 (11.1, 12.4)	12.6 (11.4, 13.5)
NE, 10^9/L	3.6 (2.8, 4.8)	3.7 (3.0, 4.6)	3.5 (2.6, 4.8)
WBC, 10^9/L	6.0 (5.0, 7.6)	6.3 (5.2, 7.4)	6.3 (4.5, 7.9)
CRP, mg/L	2.1 (1.0, 5.5)	2.1 (1.1, 6.2)	2.6 (0.8, 13.1)
ALT, U/L	38.7 (26.2, 54.2)	40.6 (27.0, 56.2)	35.0 (25.0, 55.0)
AST, U/L	35.1 (26.9, 50.2)	34.6 (27.1, 48.9)	39.0 (30.7, 56.0)
Liver cirrhosis			
absent	100 (41.2)	44 (36.1)	85 (63.9)
present	143 (58.8)	78 (63.9)	48 (36.1)
Child-Pugh			
A	231 (95.1)	115 (94.3)	132 (99.2)
B	12 (4.9)	7 (5.7)	1 (0.8)
Maximum tumor size, cm	4.8 (3.1, 7.0)	4.5 (3.2, 6.4)	5.0 (3.5, 7.0)
Tumor number			
single	211 (86.8)	106 (86.9)	105 (78.9)
multiple	32 (13.2)	16 (13.1)	28 (21.1)
Tumor differentiation			
well	26 (10.7)	7 (5.7)	24 (18.0)
moderate/poor	217 (89.3)	115 (94.3)	109 (82.0)
VETC			
negative	179 (73.7)	99 (81.1)	109 (82.0)
positive	64 (26.3)	23 (18.9)	24 (18.0)
MVI			
negative	152 (62.6)	80 (65.6)	98 (73.7)
positive	91 (37.4)	42 (34.4)	35 (26.3)
Anatomic resection			
no	178 (73.3)	84 (68.9)	101 (75.9)
yes	65 (26.7)	38 (31.1)	32 (24.1)
Surgical margin, cm			
≤1	155 (63.8)	78 (63.9)	78 (58.6)
>1	88 (36.2)	44 (36.1)	55 (41.4)
JIS score			
0	14 (5.8)	5 (4.1)	3 (2.3)
1	124 (51.0)	66 (54.1)	76 (57.1)

© 2021 Lin WP et al. *JAMA Network Open*.

2	87 (35.8)	40 (32.8)	41 (30.8)
3	18 (7.4)	11 (9.0)	13 (9.8)
TNM stage			
I	139 (57.2)	72 (59.0)	82 (61.7)
II	86 (35.4)	40 (32.8)	38 (28.6)
IIIa	18 (7.4)	10 (8.2)	13 (9.8)
BCLC stage			
0	16 (6.6)	7 (5.7)	6 (4.5)
A	202 (83.1)	102 (83.6)	101 (75.9)
B	25 (10.3)	13 (10.7)	26 (19.5)
Tokyo score			
0	14 (5.8)	4 (3.3)	1 (0.8)
1	87 (35.8)	46 (37.7)	44 (33.1)
2	100 (41.2)	49 (40.2)	42 (31.6)
3	28 (11.5)	15 (12.3)	26 (19.5)
4	11 (4.5)	6 (4.9)	9 (6.8)
5	3 (1.2)	1 (0.8)	10 (7.5)
6	0 (0)	1 (0.8)	1 (0.8)
HKLC stage			
I	126 (51.9)	64 (52.5)	64 (48.1)
IIa	0 (0)	3 (2.5)	0 (0)
IIb	105 (43.2)	48 (39.3)	53 (39.8)
IIIa	2 (0.8)	2 (1.6)	5 (3.8)
IIIb	10 (4.1)	5 (4.1)	11 (8.3)
CUPI score			
-7	153 (63.0)	71 (58.2)	75 (56.4)
-5	67 (27.6)	40 (32.8)	30 (22.6)
-4	10 (4.1)	1 (0.8)	17 (12.8)
-3	8 (3.3)	6 (4.9)	4 (3.0)
-2	5 (2.1)	4 (3.3)	3 (2.3)
-1	0 (0)	0 (0)	1 (0.8)
0	0 (0)	0 (0)	2 (1.5)
2	0 (0)	0 (0)	1 (0.8)
Recurrence			
absent	145 (59.7)	53 (43.4)	63 (47.4)
present	98 (40.3)	69 (56.6)	70 (52.6)

Continuous variables are expressed as the mean ± standard deviation (SD) or median (interquartile range [IQR]). Categorical variables are expressed as numbers (%).

Abbreviations: JIS, Japan Integrated Staging; TNM, AJCC tumor-node-metastasis staging system (eighth edition); BCLC, Barcelona Clinic Liver Cancer; HKLC, Hong Kong Liver Cancer; CUPI, Chinese University Prognostic Index; VETC, vessels encapsulating tumor clusters; MVI, microvascular invasion; HBV, hepatitis B virus; HCV, hepatitis C virus; AFP, alpha-fetoprotein; PLT, platelets; ALB, albumin; PT, prothrombin time; NE, neutrophil; WBC, white blood cells; CRP, C-reactive protein; ALT, alanine transaminase; AST, aspartic transaminase.

© 2021 Lin WP et al. *JAMA Network Open*.

eTable 2. Contributions of Different Variables to the Prediction of HCC Recurrence

Model	Training cohort				Internal validation cohort				External validation cohort			
	Comparing C-index		Likelihood ratio test		Comparing C-index		Likelihood ratio test		Comparing C-index		Likelihood ratio test	
	C-index (95% CI)	*P* value	χ^2	*P* value	C-index (95% CI)	*P* value	χ^2	*P* value	C-index (95% CI)	*P* value	χ^2	*P* value
VMNS model	0.702 (0.653-0.752)				0.673 (0.611-0.735)				0.720 (0.665-0.776)			
**Effect of adding variable to VMNS model**												
+ Tumor differentiation	0.712 (0.665-0.760)	0.155	2.574	0.109	0.675 (0.612-0.737)	0.863	0.074	0.786	0.730 (0.676-0.784)	0.228	1.357	0.244
**Effect of deleting variables from VMNS model**												
- VETC	0.696 (0.646-0.745)	0.489	7.453	0.006	0.643 (0.579-0.707)	0.027	8.190	0.004	0.704 (0.648-0.759)	0.140	5.774	0.016
- MVI	0.685 (0.633-0.737)	0.191	6.575	0.010	0.661 (0.593-0.730)	0.489	1.672	0.196	0.704 (0.644-0.764)	0.255	4.961	0.026
- (VETC and MVI)	0.672 (0.618-0.726)	0.103	16.680	<0.001	0.625 (0.555-0.696)	0.032	11.127	0.004	0.689 (0.630-0.748)	0.073	12.125	0.002

"+" indicates the addition of the variable to the VMNS model; "-" indicates the deletion of the variables from the VMNS model; $\chi 2$ is the likelihood ratio statistic for the variables when added to the VMNS model or deleted from the VMNS model.

Abbreviations: HCC, hepatocellular carcinoma; C-index, Harrell concordance index; CI, confidence interval; VMNS, VETC (vessels encapsulating tumor clusters)-MVI (microvascular invasion)-number-size; VETC, vessels encapsulating tumor clusters; MVI, microvascular invasion.

© 2021 Lin WP et al. *JAMA Network Open*.

eTable 3. 2-Year AUROC of Different Models of Recurrence-Free Survival in the Training and Validation Cohorts

Prognostic model	Training cohort		Internal validation cohort		External validation cohort	
	2-year AUROC (95% CI)	*P* value (compared with VMNS score)	2-year AUC (95% CI)	*P* value (compared with VMNS score)	2-year AUROC (95% CI)	*P* value (compared with VMNS score)
VMNS score	0.754 (0.686 - 0.822)		0.723 (0.631 - 0.815)		0.808 (0.727 - 0.889)	
TNM stage	0.668 (0.600 - 0.736)	0.001	0.627 (0.540 - 0.713)	0.005	0.710 (0.627 - 0.794)	0.003
BCLC stage	0.603 (0.551 - 0.654)	< 0.001	0.563 (0.499 - 0.627)	< 0.001	0.644 (0.569 - 0.718)	< 0.001
HKLC stage	0.653 (0.586 - 0.721)	0.003	0.643 (0.554 - 0.732)	0.030	0.706 (0.620 - 0.791)	0.020
JIS score	0.687 (0.619 - 0.755)	0.003	0.621 (0.531 - 0.711)	0.003	0.716 (0.633 - 0.799)	0.003
Tokyo score	0.637 (0.565 - 0.709)	0.001	0.610 (0.515 - 0.705)	0.008	0.684 (0.592 - 0.775)	0.009
CUPI score	0.596 (0.528 - 0.664)	< 0.001	0.498 (0.406 - 0.590)	< 0.001	0.672 (0.584 - 0.760)	0.010

Abbreviations: AUROC, area under the receiver operating characteristic curve; CI, confidence interval; VMNS score, VETC (vessels encapsulating tumor clusters)-MVI (microvascular invasion)-number-size score; JIS, Japan Integrated Staging; TNM, AJCC tumor-node-metastasis staging system (eighth edition); BCLC, Barcelona Clinic Liver Cancer; HKLC, Hong Kong Liver Cancer; CUPI, Chinese University Prognostic Index.

© 2021 Lin WP et al. *JAMA Network Open*.

eTable 4. Hazard Ratio and 2- and 5-Year Recurrence-Free Survival According to Each Risk Group Defined by the VMNS Score

Cohort	Risk group	n	HR (95% CI)	*P* value	2-year RFS, %	5-year RFS, %
Training cohort	low	122	ref		81.4	73.1
	medium	84	2.051 (1.248 - 3.371)	< 0.001	62.1	54.5
	high	37	5.465 (2.624 - 11.380)	< 0.001	30.1	18.0
Internal validation cohort	low	64	ref		63.8	57.3
	medium	42	1.876 (1.064 - 3.306)	0.009	44.2	35.5
	high	16	4.310 (1.534 - 12.110)	< 0.001	10.0	10.0
External validation cohort	low	70	ref		79.8	56.8
	medium	44	2.649 (1.470 - 4.776)	< 0.001	46.3	24.1
	high	19	6.183 (2.247 - 17.010)	< 0.001	11.1	0

Abbreviations: VMNS score, VETC (vessels encapsulating tumor clusters)-MVI (microvascular invasion)-number-size score; HR, hazard ratio; CI: confidence interval; RFS, recurrence-free survival.

© 2021 Lin WP et al. *JAMA Network Open*.

eFigure 1. Representative Morphological Features of VETC in HCC Tissues by Immunohistochemical Staining of Human CD34
(A) VETC-positive phenotype distinguished by vessels that formed a cobweb-like pattern and encapsulated tumor clusters.
(B) VETC-negative phenotype distinguished by the presence of capillary vessels.
Abbreviations: VETC, vessels encapsulating tumor clusters; HCC, hepatocellular carcinoma.

A

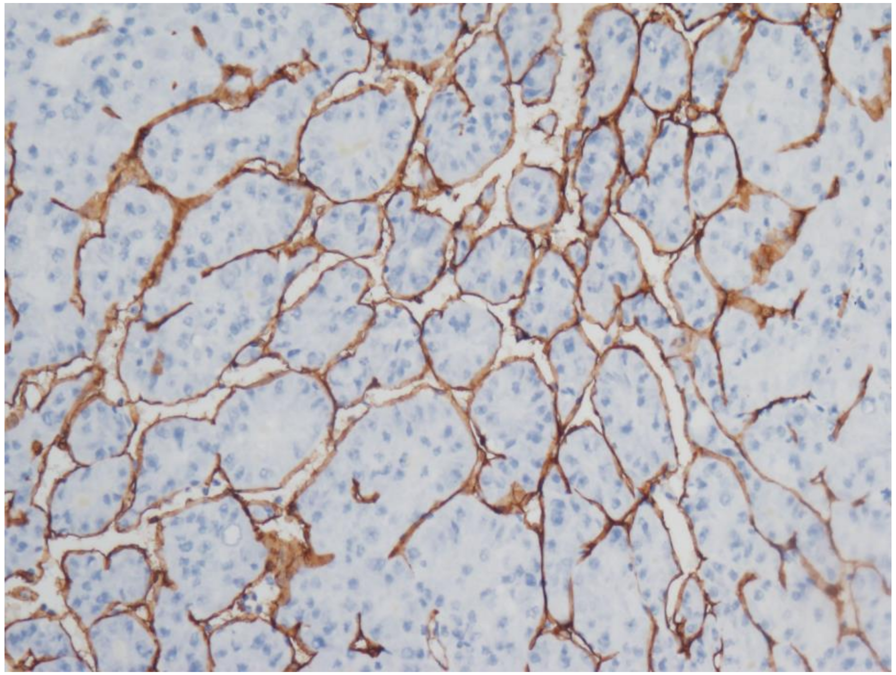

VETC-positive

B

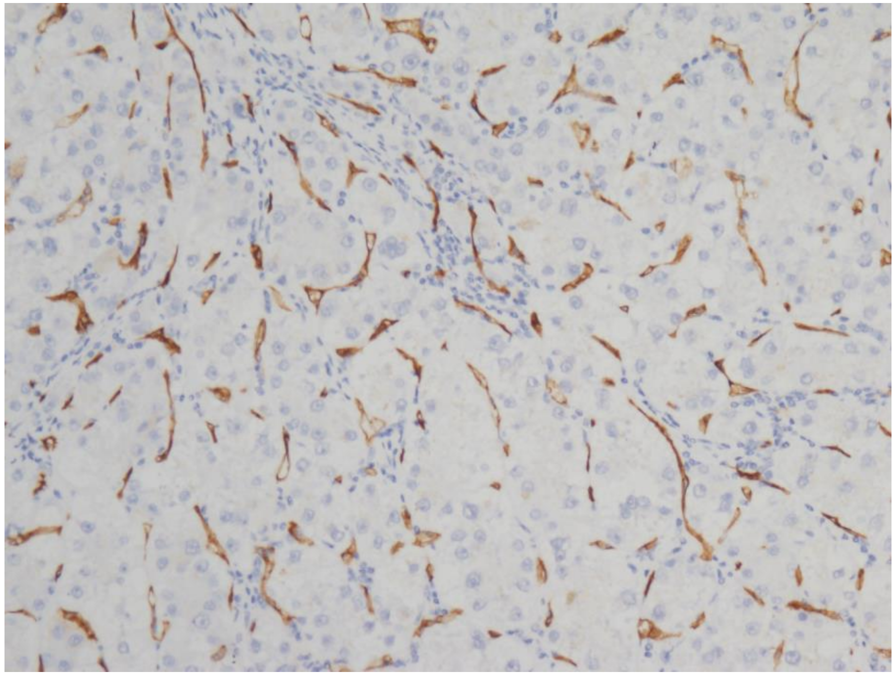

VETC-negative

© 2021 Lin WP et al. *JAMA Network Open*.

eFigure 2. Variable Selection Using LASSO Cox Proportional Hazards Regression Model

(A) The two vertical lines are drawn at the optimal values by minimum criteria (left) and 1-SE criteria (right), which result in selecting 5 variables (VETC, MVI, tumor number, ln (maximum tumor size) and tumor differentiation) and 4 variables (VETC, MVI, tumor number and ln (maximum tumor size)), respectively. Details are provided in Methods.
(B) LASSO coefficient profiles of the 23 variables. Each curve corresponds to a variable.
Abbreviations: LASSO, the least absolute shrinkage and selection operator; SE, standard error; VETC, vessels encapsulating tumor clusters; MVI, microvascular invasion.

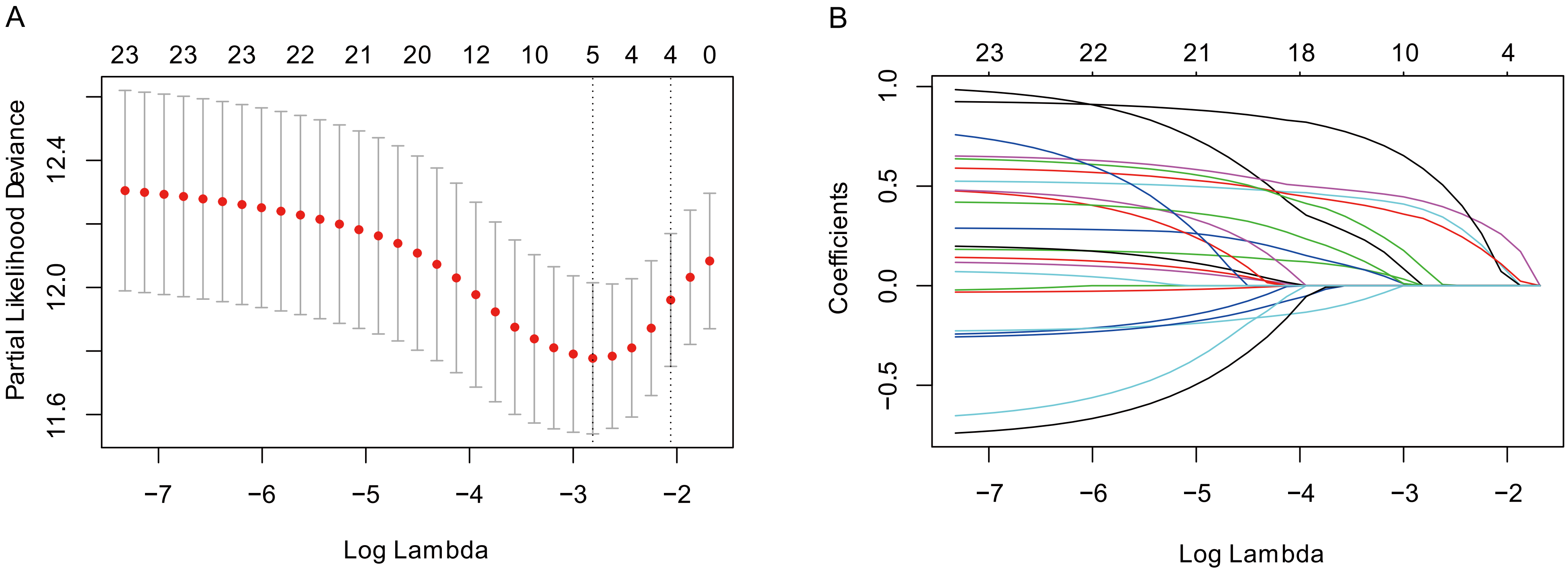

© 2021 Lin WP et al. *JAMA Network Open*.

eFigure 3. Calibration Curves of the VMNS Nomogram at 2 Years in Each Cohort

(A, training cohort; B, internal validation cohort; C, external validation cohort). Abbreviations: RFS, recurrence-free survival; VMNS, VETC (vessels encapsulating tumor clusters) -MVI (microvascular invasion)-number-size.

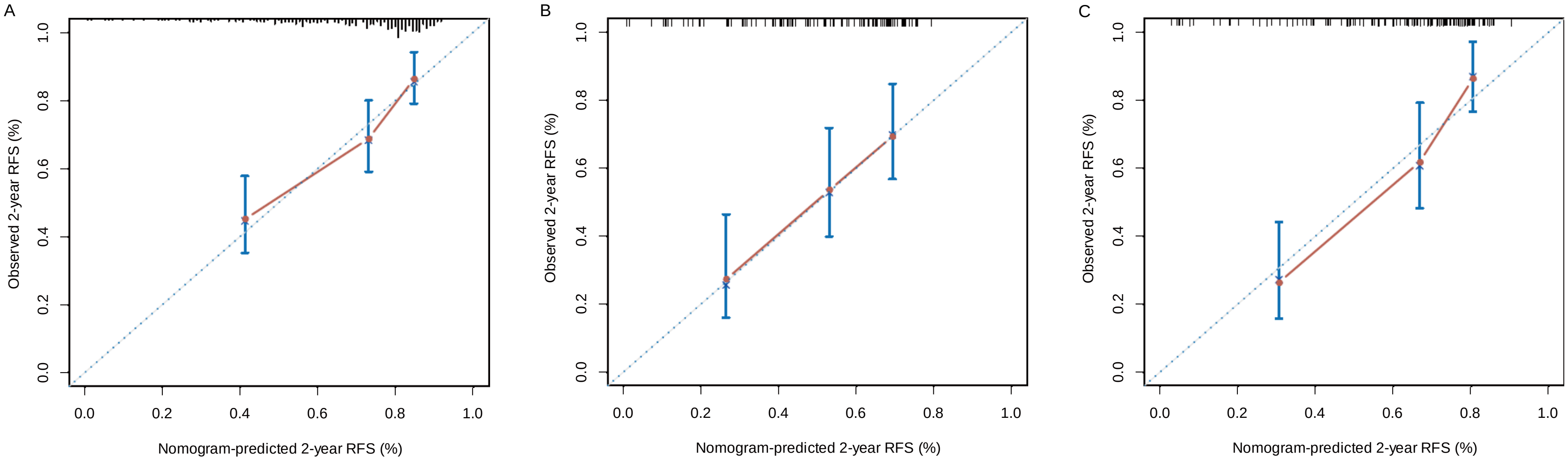

© 2021 Lin WP et al. *JAMA Network Open*.
